# Breastfeeding Practices of Ethnic Minorities in China: A Population-Based Cross-Sectional Study of 10,408 Mothers

**DOI:** 10.1007/s10903-019-00918-1

**Published:** 2019-07-19

**Authors:** Yu Zhang, Hanyu Wang, Yiqing Wang, Kun Tang

**Affiliations:** 1 School of Health Humanities, Peking University Health Science Center, No. 38 Xueyuan Rd, Haidian District, Beijing, 100084 People’s Republic of China; 2 School of Basic Medical Sciences, Peking University Health Science Center, Xueyuan Rd, Haidian District, No. 38, Beijing, 100084 People’s Republic of China; 3 Research Center for Public Health, Tsinghua University, Haidian District, Beijing, 100084 People’s Republic of China

**Keywords:** Breastfeeding practices, Ethnic minorities, China, Migration, Acculturation, Postpartum support

## Abstract

Heterogeneous characteristics of Chinese ethnic groups and their acculturation might contribute to different breastfeeding patterns. This study aimed to explore the breastfeeding practices of ethnic minorities in China considering migration and acculturation. We included 10,408 mothers from a population-based study in China. Ethnic minority was defined as ethnics other than Han. Logistic regression and adjusted prevalence were employed to analyze the association between ethnicity and breastfeeding outcomes. Both Minority group (where both parents were ethnic minorities) had a higher odds of exclusive breastfeeding and predominant breastfeeding. After stratification, this significantly higher odds of exclusive breastfeeding was solely observed among local mothers (those who never immigrated). Husbands caring for mothers “sitting the month”, which could contribute to exclusive breastfeeding, was more common among local Both Minority group. Parental ethnicities and their acculturation could influence breastfeeding practices in China. Interventions should consider ethnic differences as well as acculturation.

## Background

It is well established in previous literature that breastfeeding is beneficial for the health of mothers and the development of infants [1]. The American Academy of Pediatrics (AAP) recommended exclusive breastfeeding for the first 6 months of a child’s life, with continued breastfeeding and complementary foods at least until the infant’s first birthday [1]. The practices of breastfeeding has been a topic receiving close review in the recent years; numerous interventions at multiple levels were designed to enhance breastfeeding practices [2]. Nevertheless, the importance of breastfeeding remained unrecognized in many regions worldwide and its prevalence far from reaching an optimal level [3, 4]. According to a recent cross-sectional study conducted in 55 counties from 30 provinces in China, the proportion of infants who were ever breastfed was 79.6%, and only 20.8% of 14,539 children surveyed were breastfed exclusively until end of the first 6 months, as per AAP recommendations [5].

Research conducted by the World Health Organization (WHO) suggested that most mothers were biologically competent to breastfeed their infants, except those with severe medical conditions [6]. The practice of breastfeeding, however, was not solely determined by biological factors, but largely influenced by mothers’ socio-economic status, such as education and income [7], ethnicity [8], and immigration status [9]. Studies also revealed that social support during the postpartum period, i.e. caring and comforting, substantially influenced mothers’ breastfeeding patterns [10, 11].

Previous studies identified parents’ ethnicity as an important factor associated with the breastfeeding practice [12, 13]. Research conducted in western countries generally found that ethnic minority mothers were more likely to breastfeed their newborns [14–16]. A study conducted in the United States found that Hispanic women were more likely to ever breastfeed their infants and maintain this practice than Caucasian mothers [14]. Ladewig et al. [15] also observed that ethnic minorities in the United States, such as Chinese and African immigrants, had a higher tendency to initiate and adhere to the practice of breastfeeding. Another study conducted in the UK revealed the same pattern and noted additionally that having a life partner of ethnic minority status was a positive predictor for exclusive breastfeeding [16].

The relationship between breastfeeding practice and ethnicity became further complicated as immigration catalyzed the process of acculturation, creating more ethnic diversity. American-born Hispanic parents were less likely to breastfeed their children, compared to Hispanic immigrant parents [17]. Moreover, it was suggested that the practice of breastfeeding became less common amongst immigrants as their years of residency in the US increased [18]. As a case in point, previous studies observed that the acculturation level of ethnic minority mothers was closely associated with their breastfeeding patterns. Mothers with low levels of acculturation, in other words clinging to the values of their ethnicities, breastfed their infants more frequently and consistently than mothers with high levels of acculturation; most of minority ethnic cultures, juxtaposed to the US culture, placed an emphasis on the necessity of breastfeeding practices [18, 19].

There are 56 defined ethnic groups in China, with the group Han being the mainstream, accounting for about 91% of the national population. The other 55 ethnic groups represent the minority and account for about 9% of population in China [20]. The native minority groups in China are highly differentiated as they all have different dialects, ancestral histories and religions as they are separately isolated from one another [21]. The heterogeneity of Chinese ethnic groups could potentially exert a significant impact on breastfeeding patterns; however this topic receives little attention, few Chinese studies showed interest and the findings were generally inconsistent [22–26].

It is worth noting that China has undergone one of the largest assimilation between rural to urban regions in the last 30 years. Migration of ethnic minority groups from their native lands and villages into counties and cities has been increased greatly in the recent years [27]. Consequently, an investigation on the dynamic relation between ethnicity and breastfeeding become pressingly imperative.

This study aimed to explore the breastfeeding practices in ethnic minority groups in China, particularly, with the consideration of migration, assimilation and postpartum social support.

## Methods

### Study Design and Participants

This population-based study was conducted in 12 geographically defined regions in China. Multi-stage sampling technique was adopted for selection of participants. In the first stage, population proportionate sampling method was applied in consideration of the population structure and the status of maternal and child health and of socio-economic development. Markedly, 12 regions, including 4 megacities (Beijing in North China, Nanning of Guangxi Province in South Central China, Hefei of Anhui province and Nanjing of Jiangsu province in East China), 4 medium-sized cities (Longyan of Fujian province in East China, Liaoyuan of Jilin province in Northeast China, Honghe of Yunnan province and Luzhou of Sichuan province in Southwest China), 2 countryside areas (Zhengding County of Hebei province in North China and Jia County of Henan province in Central China) and 2 underdeveloped rural areas (Tailai county of Heilongjiang province in Northeast China and Ledu county of Qinghai province in Northwest China) were selected. In the second stage, one subregion, usually a district or county, was picked from each of the 12 regions by the method of simple random sampling. The third stage comprised of selecting 4–8 communities within each of the 12 subregions by the same sampling technique; finally a total of 77 communities and villages were included. 12 survey teams, each comprised of 5 full-time staff with medical qualification and field expertise, were sent to the 12 subregions and were asked to cover the selected communities per subregion. Mothers whose children were under 12 months were eligible for this study. The participants were provided with small gifts like soaps and wash cloth, which worth around 1 USD each, as acknowledgement for their participation. By the end, 10,408 mothers were recruited for the present study. After registration and obtaining written informed consent, trained health staff with smartphone-based questionnaires interviewed participants and recorded their response. The questionnaire surveyed mothers’ socio-demographic information, socio-economic information, breastfeeding behavior and breastfeeding related environment. Over 90% of participants completed the entire questionnaire.

This study has been approved by the Institutional Review Boards at Peking University Health Science Center and the China National Center for Disease Control. All the participants included in this study provided informed consent.

### Exposures

Parental ethnicities were the main exposures in the study. In the survey, maternal and paternal ethnicity status were collected during the interview from the interviewed mothers. For the 56 ethnic groups in China, the common 19 groups were explicitly listed and the rest classified as “others” in the survey. Since Han Chinese dominated the population with a population proportion of 91.60%, all other ethnicities were regarded as minority groups [28]. Participants were placed in Both Han group if both the mother and the husband were Han Chinese. Parents who were half Han and half ethnic minority were placed in either Maternal Minority group or Paternal Minority group. Both Minority group corresponded to both parents being ethnic minorities.

### Outcomes

Four breastfeeding outcomes were calculated, including early initiation of breastfeeding (EIB), exclusive breastfeeding infants under 6 months (EBF), predominant breastfeeding under 6 months (PBF), and children ever breastfed (Ever BF). EIB prevalence was defined as proportion of mothers with children born in the last 12 months who were put to the breast within an hour of birth (World Health Organization, 2008); Ever BF prevalence was defined as proportion of children born in the last 12 months who were ever breastfed. EBF prevalence was defined as proportion of infants 0–6 months of age who were fed exclusively breast milk; PBF prevalence was defined as proportion of infants 0–6 months of age who were predominantly breastfed, which mainly comprised of children who were fed with breast milk and water [29]. EBF and PBF were defined according to Wellstart International’s toolkit for monitoring and evaluating breastfeeding activities using a 24-h recall methodology [29]. The final calculations of EBF and PBF prevalence were computed for mothers with children from 0 to 6 months, and the calculations for EIB and Ever BF prevalence were computed for mothers with children from 0 to 12 months of age. Furthermore, the present study implemented indicator of current breastfeeding (CBF), defined as any breastfeeding in the last 24 h of children less than 12 months of age, in order to explore mothers’ current breastfeeding status [29].

### Other Covariates

Mother-infant indicators and family socio-economic status were the two categories of covariates incorporated in the present study. Mother-infant indicators included maternal age, pre-pregnancy BMI, gestational age, infant birth weight, infant sex, parity, and delivery method. Family socio-economic status included: regions, household income, residency status, and maternal occupation. These data were collected from mothers’ self-reported questionnaire. Pre-pregnancy BMI was calculated based on mothers’ self-reported weight and height before pregnancy. Regions were defined geographically by administrative level, population, and economic development, and were sorted into four categories which were megacity, urban, rural and poor rural. In the stratified analysis, megacity and urban were combined to urban; rural and poor rural were combined to rural. Household income was a continuous variable in the baseline survey, later converted into discrete data in the present analysis and sorted into ≤ 40,000 Yuan (1 USD ≈ 6.33 Chinese Yuan); 40,000–60,000 Yuan; 60,000–100,000 Yuan; and ≥ 100,000 Yuan. Occupation was divided into 4 categories: agriculture related workers, factory workers, office workers, and others. Residency status was classified as local or migrant, based on mothers’ migration history. Mothers’ health seeking behavior was regarded as another related covariate; it assessed whether breastfeeding was ever considered before pregnancy and the accessibilities to local postpartum support group and breastfeeding education. “Sitting the month”, otherwise known as Zuo Yuezi in mandarin, referred to the Chinese postpartum confinement for a period of 1 month, during which women are advised to follow several hygienic and dietary guidelines, stay indoors and recover from the delivery [30]. Postpartum supporters for mothers “sitting the month” was believed to have crucial impact; namely, the specific family member taking on such role, be it husband, maternal mother or mother in law, was examined in relation to mothers’ breastfeeding patterns.

### Data Analysis

Descriptive analyses were used to illustrate the basic demographic, socio-economic and lifestyle characteristics in different ethnic groups. Incomplete data, i.e. partially filled questionnaire, were excluded from multivariate analyses. The associations between parental ethnic groups and breastfeeding outcomes were analyzed using logistic regression model. The odds ratios were presented with 95% confidence interval. Both Han group was chosen as the reference group in all models. Two logistic models were fitted: (1) unadjusted; (2) adjusted for maternal age, infant birth weight, infant sex, parity, delivery method, household income, region (mega city/urban/rural/poor rural), residential migration status, maternal education and occupation.

Stratified analysis was employed to further explore the relationship between parental ethnicities and EBF under the effect of different covariates (region, migration status and postpartum supporters for mothers “sitting the month”) using the adjusted logistic models as described above. In order to investigate the effect of postpartum supporters for mothers “sitting the month” on EBF, two logistic models were fitted: (1) unadjusted; (2) adjusted for the same variables as listed above. Husband caring for mothers “sitting the month” was chosen to be the reference group in both models. To further explore this dynamic, the adjusted prevalences of postpartum supporters for mothers “sitting the month” in different ethnicity groups were calculated after stratifying by migration status. The adjusted variables were the same as in the logistic model mentioned above. The adjusted prevalence was based on logistic regression and the detailed method was described elsewhere [31]. All the analyses were conducted using SAS version 9.4 (SAS Institute, Cary, North Carolina, USA).

## Results

The baseline characteristics of the study population were assorted by parental ethnicities and displayed in Table 1. Of all 10,346 participants included in the analysis, 8479 (81.95%) with a mean age of 29.36 (SD = 4.95) were placed in Both Han group, 569 (5.50%, mean age of 28.96 with SD = 4.95) placed in Maternal Minority group, 426 (4.12%, mean age of 29.40 with SD = 5.12) placed in Paternal Minority group, and 872 (8.43%, mean age of 29.40 with SD = 5.21) placed in Both Minority group. The distributions of baseline covariates, including pre-pregnancy BMI, gestational age, infant birth weight and infant sex, were similar across all parental ethnic groups, with a slightly higher proportion of male infants in Maternal Minority group (55.18%). Overall, more women in Both Han group were observed to have cesarean section (40.92%), higher household income, college and above education (59.13%), office-based careers (49.49%), and no history of migration (63.25%). Women in Both Minority group tended to have vaginal delivery (58.49%), an annual household income of less than 40,000 Yuan (63.29%), primary school and below education (31.31%), agriculture-related occupation (51.72%) and a history of migrating to urban areas (54.82%); it was also noted that they tended to be multiparous. Overall, the EIB prevalence were higher in Both Han (72.43%) and Both Minority groups (72.94%), in comparison to the EIB prevalence in Maternal Minority (64.67%) and Paternal Minority groups (68.08%). The prevalence of CBF and Ever BF were similar across four groups. EBF prevalence was found to be a more prevalent practice in Both Minority group (22.41%). Health seeking behaviors were analyzed and we observed in the Both Minority group that a smaller proportion of mothers who attended mother support group (12.39%), or considered breastfeeding early (32.03%), or received breastfeeding education (46.10%) or went to postpartum caring centers (1.49%), compared with other groups.

**Table 1:** Basic characteristics

	Parental ethnicities			
	Both Han N = 8479	Maternal Minority N = 569	Paternal Minority N = 426	Both Minority N = 872
Maternal age, year (SD)	29.36 (4.95)	28.96 (5.12)	29.40 (5.21)	27.15 (6.08)
Pre-pregnancy BMI, kg/m^2^ (SD)	22.18 (4.17)	21.45 (3.90)	22.12 (4.85)	21.50 (4.04)
Gestational age, week (SD)	39.03 (1.37)	38.94 (1.56)	38.90 (1.38)	38.73 (1.67)
Infant birth weight, kg (SD)	3.38 (0.67)	3.29 (0.61)	3.34 (0.69)	3.15 (0.50)
Infant sex (%)				
Male	50.24	55.18	52.82	51.95
Female	49.76	44.82	47.18	48.15
Parity (%)				
Primiparous	45.83	50.26	52.35	40.60
Multiparous	54.17	49.74	47.65	59.40
Delivery method (%)				
Vaginal	51.51	52.46	49.30	58.09
Instrument assisted delivery	7.58	11.97	11.97	16.76
Cesarean section	40.92	35.56	38.73	25.14
Region (%)				
Mega city	33.26	44.82	50.00	35.09
Urban	30.10	34.80	33.57	54.82
Rural	19.67	2.28	2.11	0.80
Poor rural	19.67	18.10	14.32	9.29
Household income (%)				
≤ 40,000 yuan	22.92	36.95	32.88	63.29
40,001–60,000 yuan	23.93	23.39	21.00	17.86
60,001–100,000 yuan	31.48	26.44	30.59	13.69
> 100,000 yuan	21.67	13.22	15.53	5.16
Highest education (%)				
Primary school and below	5.25	11.95	6.57	31.31
High school	35.62	30.05	36.15	43.46
College and above	59.13	58.00	57.28	25.23
Occupation (%)				
Agriculture related	15.77	20.74	18.59	51.72
Factory workers	16.15	14.06	11.29	7.68
Clerk	49.49	42.36	49.18	22.36
Others	18.59	22.85	20.94	18.23
Resident status (%)				
Local	63.25	56.41	49.77	54.13
Migrant	36.75	43.59	50.23	45.87
Breastfeeding practice (%)				
Early initiation	72.43	64.67	68.08	72.94
Current BF	87.16	85.24	85.45	87.73
Ever BF	97.48	97.01	98.12	97.94
Exclusive BF (0–6 months)	14.96	14.10	11.30	22.41
Health seeking behavior (%)				
Attending supporting group	37.54	32.16	37.56	12.39
Considering breastfeeding early	51.24	41.20	39.15	32.03
Receiving breastfeeding education	56.59	52.20	51.88	46.10
Going to postpartum caring center	8.00	9.31	10.33	1.49

The relationship between breastfeeding practices and parental ethnicities was presented in Table 2. No significant association was found in terms of CBF and Ever BF across the 4 groups. In the fully adjusted model, a lower odds of EIB was found in Maternal Minority (OR: 0.67, 95% CI 0.52–0.87) and Paternal Minority group (OR: 0.73, 95% CI 0.54–0.99), compared to Both Han group. Mothers in the Both Minority group had a significantly higher tendency of EBF (OR: 1.78, 95% CI 1.15–2.76) and PBF (OR: 2.08, 95% CI 1.44–3.00), as opposed to mothers in Both Han group. The detailed ORs and 95% CIs were shown in Table 2.

**Table 2:** Relations between breastfeeding practices and parental ethnicities

	Both Han	Maternal Minority	Paternal Minority	Both Minority
Unadjusted OR (95% CI)				
Early initiation (EIB)	1	0.63 (0.48, 0.83)	0.69 (0.50, 0.94)	1.12 (0.85, 1.46)
Current BF (CBF)	1	0.91 (0.65, 1.28)	0.92 (0.62, 1.35)	1.23 (0.87, 1.74)
Ever BF (Ever BF)	1	0.84 (0.51, 1.39)	1.35 (0.66, 2.76)	1.23 (0.76, 2.00)
Exclusive BF (EBF) (0–6 months)	1	0.93 (0.67, 1.30)	0.73 (0.48, 1.10)	1.64 (1.29, 2.10)
Predominant BF (PBF) (0–6 months)	1	0.99 (0.77, 1.28)	0.68 (0.50, 0.93)	1.88 (1.53, 2.31)
Adjusted OR (95% CI)^a^				
Early initiation (EIB)	1	0.67 (0.52, 0.87)	0.73 (0.54, 0.99)	1.38 (1.08, 1.77)
Current BF (CBF)	1	0.72 (0.51, 1.00)	0.72 (0.47.1.06)	0.94 (0.66, 1.32)
Ever BF (Ever BF)	1	0.62 (0.33, 1.17)	1.98 (0.61, 6.38)	0.99 (0.46, 2.14)
Exclusive BF (EBF) (0–6 months)	1	1.19 (0.73, 1.93)	0.72 (0.35, 1.47)	1.78 (1.15, 2.76)
Predominant BF (PBF) (0–6 months)	1	1.20 (0.82, 1.78)	0.86 (0.52, 1.42)	2.08 (1.44, 3.00)

^a^Adjusted for maternal age, infant birth weight, infant sex, parity, delivery method, household income, region (super city/urban/rural/poor rural), residency (local/migrant), occupation, and education

It was worth noting that the significantly higher odds of EBF observed in Both Minority group was solely observed among mothers who lived in local areas, as shown in Table 3 (OR: 1.85, 95% CI 1.05–3.26). This trend in Both Minority group disappeared however, after stratified by areas (rural and urban) and postpartum supporters for mothers “sitting the month”.

**Table 3:** Relations between exclusive breastfeeding and ethnicity in different socio-economic groups

	Both Han	Maternal Minority	Paternal Minority	Both Minority
Region				
Urban	1	1.03 (0.56, 1.91)	0.66 (0.28, 1.54)	1.40 (0.82, 2.40)
Rural	1	1.11 (0.45, 2.76)	0.55 (0.12, 2.46)	0.88 (0.17, 4.45)
Resident status				
Local	1	1.49 (0.85, 2.62)	0.80 (0.32, 1.97)	1.85 (1.05, 3.26)
Migrant	1	0.32 (0.09, 1.08)	0.40 (0.12, 1.37)	0.81 (0.39, 1.71)
Postpartum supporters for mothers “sitting the month”				
Husband	1	0.73 (0.31, 1.72)	0.43 (0.13, 1.37)	1.09 (0.50, 2.38)
Maternal mother	1	1.17 (0.45, 3.04)	1.00 (0.28, 3.57)	0.99 (0.36, 2.73)
Mother in law	1	1.22 (0.51, 2.88)	0.25 (0.03, 1.89)	1.79 (0.92, 3.48)

The adjusted variables are other variables in the table and maternal age, pre-pregnancy weight, gestational age, infant birth weight, and infant sex

Moreover, the association between EBF and postpartum supporters for mothers “sitting the month” was examined and presented in Table 4. Mothers who were cared by maternal mother (OR: 0.78, 95% CI 0.65–0.93) and mother in law (OR: 0.73, 95% CI 0.62–0.86) had a significantly lower odds of EBF, compared to mothers “sitting the month” cared by their husband in the fully adjusted model.

**Table 4:** Association between exclusive breastfeeding and different postpartum supporters for mothers “sitting the month”

	Total sample		
	EBF rate (%)	Crude model	Adjusted model^a^
Postpartum supporters for mothers “sitting the month”			
Husband	32.8	1	1
Maternal mother	29.0	0.83 (0.70–0.99)	0.78 (0.65–0.93)
Mother in law	27.2	0.76 (0.66–0.89)	0.73 (0.62–0.86)
Others	33.7	1.04 (0.81–1.33)	0.86 (0.67–1.12)

^a^Adjusted for maternal age, infant sex, region, maternal education, maternal job, delivery method and parity

Adjusted prevalence of postpartum supporters for mothers “sitting the month” in different parental ethnic groups were calculated, after stratified by migration status, and presented in Table 5. In Both Minority group, mothers with no migration history (adjusted prevalence: 43.11%, 95% CI 38.07–48.15%) were more likely to be cared by their husband than migrant mothers (OR: 28.42%, 95% CI 25.21–31.63%). Postpartum supporters for mother “sitting the month*”* were similar in migrated Both Minority group and Both Han group.

**Table 5:** Adjusted prevalence (95% CI)* of postpartum supporters for mothers “sitting the month” in different parental ethnicity groups, stratified by migrant status

Postpartum supporters for mothers “sitting the month”	Resident status	Both Han	Maternal Minority	Paternal Minority	Both Minority
Husband	Local	17.98 (16.28, 19.68)	27.38 (22.64, 32.12)	25.70 (20.84, 30.56)	43.11 (38.07, 48.15)
	Migrant	14.87 (12.88, 16.86)	20.76 (19.41, 22.11)	33.63 (24.94, 42.33)	28.42 (25.21, 31.63)
Maternal mother	Local	29.25 (27.20, 31.30)	22.90 (17.99, 27.81)	43.07 (38.70, 47.44)	11.06 (7.26, 14.85)
	Migrant	26.28 (23.77, 28.78)	29.42 (14.51, 44.33)	31.68 (23.49, 39.88)	20.62 (14.50, 26.73)
Mother in law	Local	46.60 (44.33, 48.86)	28.22 (25.28, 31.16)	25.22 (17.24, 33.20)	44.69 (39.80, 49.58)
	Migrant	49.63 (46.96, 52.29)	33.21 (20.35, 46.08)	22.37 (19.32, 25.42)	50.17 (43.76, 56.58)

*After adjusted for household income, education, maternal age, pre-pregnant weight, infant sex, infant weight, gestational age, and rural/urban

## Discussion

The current study presented three main findings. First, infants born to ethnic minority parents were more likely to be breastfed predominantly and exclusively, compared to infants from Han families. Second, the higher prevalence of EBF in Both Minority group was solely apparent in those with no history of migration. Third, postpartum supporters for mothers “sitting the month” were similar between migrated mothers of Both Minority group and mothers of Both Han group, potentially contributing to a lower EBF prevalence in migrated Both Minority group.

As per our first finding, infants with both parents from ethnic minority groups were more likely to be exclusively and predominantly breastfed. The finding was consistent to some studies conducted in China. Namely, the mothers in Xinjiang including Kazakh, Hui and Xibe were more likely to breastfeed their infants, compared to mothers of Han ethnicity [32]. Another study found that Chinese Ewenke ethnic group tended to exclusively breastfeed their infants, potentially due to their adherence to traditional practice and understandings of the breastfeeding benefits to both mothers and infants [33]. Conversely, some studies revealed contrary results. A study observed that Uygur mothers were less likely to exclusively breastfeed their babies. One potential interpretation was their particular ethnic belief of breastmilk being insufficient for the growth of infants [32]. In addition, mothers of Tibet ethnic group tended to exclusively breastfeed their infants for a shorter period of time, compared to the WHO guideline; this could be explained by Tibetans’ traditional feeding practice of adding water to the breastmilk [34]. The present study classified all 55 ethnic minorities in China into a single group and revealed that overall EBF was a common practice. We postulated that the overall practices of breastfeeding among ethnic minorities in China encouraged exclusive breastfeeding, compared to majority Han ethnicity. Moreover, mothers from ethnic minority groups were observed to be more likely to have vaginal delivery and be in agriculture related occupation, both contributing to a higher breastfeeding prevalence [35, 36]. This postulation was also consistent with findings from western countries, where family of ethnic minorities generally adhered to traditional practices and had a higher tendency to breastfeed than Caucasian mothers [15, 16].

The higher prevalence of EBF in Both Minority group was solely apparent in those with no history of migration. This result was consistent with previous findings. Gibson–Davis et al. [37] found that each additional year of US residency decreased the odds of breastfeeding by 4% for foreign-born mothers. Another study observed that Asian mothers living in UK would have breastfed for longer period of time if their baby had been born in the mother’s country of birth [38]. Acculturation to the dominant culture with less breastfeeding practices, which happened with immigration, might have detrimental impact on breastfeeding practices of ethnic minority groups [17]. We postulated that migrated ethnic families, mostly inhabiting in urban and other Han dominated areas, were likely to abandon their traditional breastfeeding practice and adopt Han’s disfavor of breastfeeding. Furthermore, it would be hard for migrant ethnic minority mothers to find ethnic communities in the urban city, detaching them from their traditional values and beliefs [8, 16]. At a stroke, acculturation into the dominant culture and lack of support from ethnic family and communities contributed to the lower breastfeeding prevalence in migrated ethnic minorities. Besides, as we shall illustrate next, postpartum supporters, an influencing factor for breastfeeding literature [39, 40], were also changed in migrated minorities, which might explain the acculturation of ethnic minorities.

Interestingly, a break of postpartum supporters for mothers “sitting the month” was observed in migrated ethnic minorities with reference to local ethnic minorities. The variation could potentially explain the previously discussed finding. It was revealed to us during the study that a significant higher odds of EBF was apparent when husband cared for their wives “sitting the month”; the opposite was observed when maternal mothers and mothers-in-law undertook the role. In the dominant Han culture, it was common for maternal mothers and mothers-in-law to clean and care for mothers “sitting the month”, as was observed in our study. Immigration of ethnic minorities would potentially result in the assimilation of Han’s “sitting the month” care custom, consequently contributing to a lower exclusive breastfeeding pattern for the migrated ethnic minorities.

Based on a large-scale population-based survey in China, the present study had a large sample size ensuring the precision of our results. This study added to the scant literature available, exploring the relationship between ethnicity and breastfeeding practices in China. Admittedly, there were several limitations in this study. First, the present study combined different ethnicities into one group and compared them with majority Han people. Caution should be taken that this general finding might be unsuitable to some specific ethnic groups, due to the large heterogeneity in terms of culture, lifestyles, and religious practices. Future studies should be conducted in different ethnic groups, examining their ethnicity-specific breastfeeding practices respectively, and coming up with innovative solutions to the acculturation. Furthermore, to fully understand the reasons behind the different breastfeeding behaviors among various ethnic groups in China, cultural, anthropological, behavioral and sociological studies using both quantitative and qualitative methods should be conducted in the future. Second, causal inference between ethnic minorities’ residential status and breastfeeding practices was difficult to establish due to the nature of the cross-sectional study. Further perspective studies were needed in order to establish a more confident causal relationship. Third, the models used in this study might suffer from omitted variable bias. Although a broad set of characteristics was included during the data collection, there might still be other aspects, especially cultural and psychosocial factors, that were failed to consider. Future research should consider additional factors related to breastfeeding practices. Fourth, the ‘last-24-hour method’ was used to determine breastfeeding practices, which tended to overestimate the prevalence of EBF as compared to the ‘since birth method’ [41]. Last, breastfeeding outcomes were based on mothers’ own reports; this potentially created recall bias introducing systematic errors.

## Conclusions

Breastfeeding practices were different between ethnic groups in China with minorities being more likely to breastfeed their infants predominantly and exclusively. The tendency to exclusively breastfeed, however, was no longer observed in ethnic minority migrants, suggesting a potential acculturation to the Han ethnic group. Evidently, interpretation of such acculturation was elaborated, with postpartum supporters for mothers “sitting the month” being an impactful determinant. Routine and successful breastfeeding is crucial for infants’ developmental health and is largely influenced by social factors. This study emphasized the influence of parental ethnicities on breastfeeding practices. Findings from this study contributed to the growing body of research on social determinants of breastfeeding. In addition, the study added evidence to the impacts of cultural assimilation on ethnic minorities’ health in China. Future interventions, aiming at promoting breastfeeding practices, should account for the discrepancy of breastfeeding patterns between the Han group and the ethnic minorities, as well as the acculturation of ethnic minorities to the Han group.
